# Prediction of BMI traits in the Chinese population based on the gut metagenome

**DOI:** 10.1186/s12934-023-02255-3

**Published:** 2023-12-08

**Authors:** Yu Liang, Shujie Dou, Guangzhong Zhao, Jie Shen, Guangping Fu, Lihong Fu, Shujin Li, Bin Cong, Chunnan Dong

**Affiliations:** 1 College of Forensic Medicine, Hebei Key Laboratory of Forensic Medicine, Hebei Medical University, Shijiazhuang, 050017 Hebei China; 2 Department of Pathogen Biology, Hebei Medical University, Shijiazhuang, 050017 Hebei China

**Keywords:** SNP, Gut microbiota, Metagenome, Forensic science, Individual characterization

## Abstract

**Background:**

Identifying individual characteristics based on trace evidence left at a crime scene is crucial in forensic identification. Microbial communities found in fecal traces have high individual specificity and could serve as potential markers for forensic characterization. Previous research has established that predicting body type based on the relative abundance of the gut microbiome is relatively accurate. However, the long-term stability and high individual specificity of the gut microbiome are closely linked to changes at the genome level of the microbiome. No studies have been conducted to deduce body shape from genetic traits. Therefore, in this study, the vital role of gut bacterial community characteristics and genetic traits in predicting body mass index (BMI) was investigated using gut metagenomic data from a healthy Chinese population.

**Results:**

Regarding the gut microbial community, the underweight group displayed increased α-diversity in comparison to the other BMI groups. There were significant differences in the relative abundances of 19 species among these three BMI groups. The BMI prediction model, based on the 31 most significant species, showed a goodness of fit (R^2^) of 0.56 and a mean absolute error (MAE) of 2.09 kg/m^2^. The overweight group exhibited significantly higher α-diversity than the other BMI groups at the level of gut microbial genes. Furthermore, there were significant variations observed in the single-nucleotide polymorphism (SNP) density of 732 contigs between these three BMI groups. The BMI prediction model, reliant on the 62 most contributing contigs, exhibited a model R^2^ of 0.72 and an MAE of 1.56 kg/m^2^. The model predicting body type from 44 contigs correctly identified the body type of 93.55% of the study participants.

**Conclusion:**

Based on metagenomic data from a healthy Chinese population, we demonstrated the potential of genetic traits of gut bacteria to predict an individual’s BMI. The findings of this study suggest the effectiveness of a novel method for determining the body type of suspects in forensic applications using the genetic traits of the gut microbiome and holds great promise for forensic individual identification.

**Supplementary Information:**

The online version contains supplementary material available at 10.1186/s12934-023-02255-3.

## Introduction

Height, weight, and body type are important characteristics for identifying individuals in forensic science, as they help to narrow down suspects and provide clues and a basis for determining culpability [1]. BMI is an internationally recognized measure of human body type, calculated by dividing weight (kg) by the square of height (m^2^), expressed as kg/m^2^. Because it visually represents an individual’s body shape, it is sometimes used to describe the appearance of criminal suspects. Body type is related to height, weight, bone structure, and other factors. Forensic anthropology and forensic genetic approaches can be used to indirectly indicate a person’s body type by estimating their height and weight. Forensic anthropology methods determine height through regression equations derived from direct measurements or CT scans of the long bones of the upper and lower extremities, sternum, and vertebrae [2–4]. As an example, Giurazza and colleagues [2] employed a regression equation containing three variables (femur length, cranial base length, and distance from the posterior cranial base to the inferior nasal bone point) to generate the most accurate inference, and it had an average absolute error value (E%) of 2.9%. The regression equation with a single variable (femur length) had an E% of 3.2%. However, the fact that the skull and femur may not be discovered concurrently makes the one-way regression equation more applicable in practical applications. Weight was estimated using different equations based on femur dimensions, double iliac width, and height, as described in previous studies [5–7]. The weight prediction errors of the methods displayed a strong correlation with individual BMI: the weight prediction error was 16.7% when the entire sample was considered, whereas it was reduced to 9.6% when only individuals with normal BMI were considered. Morphometric methods for estimating height and weight, as discussed earlier, may involve subjective factors and require prior measurement of the skeletal information of the subject, which has some limitations in forensic practice. Forensic genetics employs SNP loci related to height to formulate models for predicting it. In their study, Liu et al. [8] constructed a prediction model for height using 180 height-related SNPs, which displayed a moderate accuracy range (AUC = 0.75, R^2^ = 0.12). Subsequently, a more accurate prediction model was obtained by using 689 height SNPs (AUC = 0.79, R^2^ = 0.21). The results of the study indicated that an increase in the number of height-related SNP loci led to an improvement in the accuracy of the height prediction model. In their study, Jiao et al. [9] utilized 547 height-related SNP loci from a European population to develop a height prediction model for the Chinese population. This model, with an AUC of 0.67, was effective in predicting the height of Han Chinese men in northern China. However, there is currently no relevant research in forensic anthropology and forensic genetics where body type was directly quantified.

Dietary factors, in addition to genetic factors [10], mainly influence body type. Therefore, predicting personal characteristics solely based on morphology and genetics has limitations. Forensic microbiology has garnered attention from forensic scientists as an emerging field in recent years. Changes in the gut microbiome may offer new opportunities for predicting personal body type. A previous study [11] showed that dietary habits influence the gut microbiota, which in turn affects obesity phenotypes. Numerous studies have also suggested that obesity is influenced by genetic factors [12] and personal factors such as gender, age, ethnicity [13], stress [14], and dietary habits [10]. However, of all these factors, dietary habits are deemed a significant and indispensable influence. Hence, the gut microbial community may play a mediating role in the relationship between diet and obesity phenotypes. A large-scale study [15] confirmed differences in gut microbiota composition, function, and ecological networks in relation to BMI. The study showed that the underweight group had significantly higher α-diversity of the bacterial community than the other BMI groups. Additionally, obese individuals were found to have an increased susceptibility to the enrichment of butyrate-acetoacetate CoA-transferase. These findings suggest a close relationship between the gut microbial community and body type. It may be possible to predict body type based on gut microbial community structure. Wang et al. [16] confirmed the forensic application of predicting body type based on gut bacteria using 16 S rRNA sequencing technology to assess microbiota diversity in healthy adults. They also developed a linear regression model that accurately predicted individual body types with 74% accuracy. The applicability of this study to forensic practice is limited by the inclusion of only 54 volunteers, all local students between the ages of 20 and 30 living in the same school in the Chengdu area. The abovementioned studies focused on the structure and abundance of microbial communities. However, changes at the microbial genome level have also been shown to be closely associated with individual characteristics.

Metagenomic sequencing allows for a thorough examination of microbial composition, abundance, gene function, and metabolic pathways in various habitats. Compared to 16 S rRNA gene sequencing, metagenomic sequencing increases sequencing depth, comprehensiveness, and accuracy and has important benefits for identifying microbial species and mining genomic data [17]. According to Chen et al. [18], metagenomic sequencing data revealed that the genetic characteristics of the gut microbiota are stable over time and highly specific to individuals. This information can be used to create a host “microbial fingerprint” and correctly classify fecal samples with 85% accuracy four years apart using genetic traits such as gut microbial SNP haplotypes and DNA sequence structure variances (SVs). In the same vein, Schloissnig et al. [19] showed that genetic characteristics of the microbiota can serve as “microbial fingerprints” of individuals. By analyzing samples from the same individual at varying times of the year, SNP patterns in the human gut were found to be relatively stable over time, whereas the structure and abundance of the microbial community were not. The findings suggest that the stability, specificity, and predictability of individual traits are greater at the gene level of gut microbial traits than traits related to microbial community diversity.

This study included adults from different provinces in China aged between 21 and 72 years to investigate the association between individual BMI and gut microbial characteristics using second-generation gut metagenomic sequencing data. The aims of this study were to develop a population BMI prediction model and a body type estimation model based on various indicators, such as the density of bacterial SNPs, and to provide a novel method for predicting the BMI characteristics of suspects in criminal investigations for practical forensic applications.

## Materials and methods

### Selection and retrieval of data

A literature search was performed using the PubMed [20] database. The search was limited to research articles written in English on healthy Chinese populations with freely available full text between June 2012 and June 2022, using the following terms: ‘metagenome,‘ ‘obesity,‘ and ‘gut,‘ and excluding literature reviews. To minimize systematic errors prior to analyzing the data, we chose fecal metagenomic data that were sequenced exclusively on the Illumina platform. This approach allowed us to amalgamate all available metagenomic data for analysis. Raw data from three studies in the NCBI database were aligned based on sequence and retrieved with corresponding phenotype information mentioned in the literature. The accession numbers of the papers used in the study included PRJNA539850, SRA045646, and PRJEB6997. We downloaded the raw sequencing data from the NCBI database using SRA Toolkit (v3.0.0) software for further analysis [21].

### Processing of metagenomic sequencing data

The analysis protocol used in this study was constructed based on prior research [22]. All metagenomic data from the three cohorts were processed together as a single dataset.

1. The SRA file (raw sequencing data) format was converted to the Fastq file format using parallel-fastq-dump (v2.11.0) with the following parameters: -t 12 -O./--split-3 --gzip.
2. FastQC (v0.11.9) was used to assess the quality of the metagenomic data [23] and establish data quality control protocols. Fastp (v0.23.2) was used for quality control [24]. First, each segment of the sequence was at least 45 bases in length, and the average quality of the sequence was not below 20. Bases that had a mass fraction of less than 20 were removed, starting from the 3’ end. A 5-mer sliding window was applied, and if the average quality of the window fell below 10 at a specific position, that section of the sequence was eliminated (parameters: -l 45 -q 20 -e 20 -W 5 -r 10).
3. Since there are no standard microbial reference genomes currently available, we constructed reference genomes for sequence alignment. MetaPhlAn (v3.1.0), based on approximately 17,000 reference genomes [25], was used to annotate the bacterial species in each sample. The genome sequences of these microbial species were downloaded from the NCBI genome database using ncbi-genome-download (v0.3.1) and then combined to form the reference genomes for this study.
4. BWA-MEM (v0.7.17) was used to align each sample sequence to the reference genome. We only kept the unique alignments [26] (parameters not labeled indicate that the software runs according to the default parameters).
5. SAMtools (v1.15.1) was used to convert alignment files in SAM format to BAM format [27], and Picard was used for annotation and filtering [28]. The parameters employed for SAMtools and Picard are “view -q 1 -bS” and “MarkDuplicates REMOVE_DUPLICATES = true MAX_FILE_HANDLES_FOR_READ_ENDS_MAP = 65000 VALIDATION_STRINGENCY = LENIENT”, respectively.
6. BCFtools (v1.15.1) was used to retrieve SNPs and determine their number in all samples, and VCFTools (v0.1.16) was used to filter the data to obtain high-quality SNPs [29]. Indel sequences were skipped, and data regarding ineligible loci were removed. The data were filtered based on the following criteria: minimum depth of coverage of 10, minimum of 4 reads supporting mutations, minimum quality score of 15, and minimum comparison quality of 15. The parameters employed for BCFtools and VCFTools were “-vm -Oz -V indels” and “-H -f +/d = 10/a = 4/Q = 15/q = 15”, respectively.

### Bioinformatics analysis and visualization

We conducted all statistical analyses in the Python (v3.11.2) and R (v4.2.2) environments. We expressed changes at the gut microbial community level as the relative abundance of species and changes at the gene level as the SNP density (number of SNPs per base per kilobase pair) of overlapping clusters (contig). After applying the SNP search method, we obtained multiple VCF format files, with each file representing a sample that included the contig length and the number of SNPs on the contig. To calculate the total SNP density of all contigs in each sample and the SNP density of each contig, we employed Python and used the SNP density formula: SNP density = (N × 1000)/L, where N represents the number of SNPs on the contig and L represents the length of the contig. We used R scripts to estimate α-diversity (species and genetic diversity within a sample) and β-diversity (species and genetic diversity between samples). Shannon (evenness estimation) and Ace (richness estimation) indices were calculated, and box plots were plotted using the ‘vegan’ R package. Furthermore, the Wilcoxon test was utilized to assess differences between groups. The analysis of our samples’ beta diversity was conducted using the Bray‒Curtis distance. We used an analysis of similarities (Anosim) to evaluate whether there were any noteworthy dissimilarities between groups. These dissimilarities were visualized using principal coordinate analysis (PCoA) plots. We also used the Spearman correlation test to assess the association between SNP density and individual BMI. Benjamini‒Hochberg (BH) was employed for correcting multiple testing to control for the false discovery rate (FDR). We considered a corrected p value < 0.05 to be statistically significant. We used the R package “tidyverse” to compare the relative abundance of species and SNP density of genes in each study group.

The random forest (RF) is a robust machine learning technique [30]. Using RF (the “randomForest” package of R), the relative abundance of taxa in the gut microbiota of healthy populations was classified and regressed against their actual BMI. The top 500 species with the highest sum of relative abundance were selected as indicators for building the model. After that, the SNP density of contigs in the gut microbiota of healthy populations was classified and regressed against their actual BMI. The top 500 contigs with the highest correlation rankings were selected as indicators to build the prediction model. During 100 iterations of the RF model, bacterial taxa and contigs were ranked based on feature importance. The number of biomarkers was determined by 10-fold cross-validation using the rfcv() function to minimize the model error. Based on the generated biomarkers, RF regression and classification models were constructed. Model performance for training and testing was determined using mean absolute error (MAE) and R^2^.

## Results

### Overview of research data

The characteristics of the studies included in Table 1, published between 2012 and 2022, are presented. The participants were from various provinces in China, and a total of 308 independent fecal samples were generated for gut metagenomic analysis. After data analysis, we obtained the species annotation files and VCF files of 308 samples, which included information on the name and relative abundance of each species (Additional file 2), as well as the number and length of contigs for the 735 species. The sample ID and corresponding BMI information for the 308 case samples are also shown in Additional file 2. We calculated the SNP densities of contigs using Python for all samples and obtained a total of 81,881 contigs (Additional file 3). Of these, 767 had a statistically significant correlation with individual BMI. For subsequent analyses, we selected the top 500 species with the highest relative abundance sums as well as the top 500 contigs with the strongest correlations. Participants were categorized into three groups according to their BMI [16]: LW (underweight): BMI < 18.5 kg/m^2^, NW (normal weight): 18.5 kg/m^2^ ≤ BMI < 24.0 kg/m^2^, and HW (overweight): BMI ≥ 24.0 kg/m^2^.

**Table 1:** Overview of data included in the study

Study ID	Age(years) Mean ± SD	Sex	BMI (kg/m^2^) Mean ± SD	Stool Sample Collection Method and Storage	Sequencing Platform
Zeng2021 [31] DOI:10.1099/mgen.0.000639	*44.7 ± 9.1*	93♀ 57♂	27.44 *±* 2.25	Collected from sterile stool containers and frozen at -80℃ ^(1)^	Illumina
Zhang 2015 [32] DOI:10.1038/nm.3914	42.9 *±* 8.4	49♀ 20♂	23.62 *±* 3.53	Collected in hospital and stored at -80℃ ^(2)^	Illumina
Qin 2012 [33] DOI:10.1038/nature11450	40.8 *±* 12.8	46♀ 43♂	21.23 *±* 3.17	Collected at home and frozen − 80℃ ^(3)^	Illumina

(1). During physical examination, fresh stools were collected from individuals using sterile stool containers. Approximately 5 g stool from each individual was obtained using swabs (Huachenyang Technology). The stool samples were preserved in stool collection tubes (Axygen), and then transferred to a − 80 °C refrigerator (DW-86L626; Haier) within half an hour.

(2). Fecal samples were collected at Peking Union Medical College Hospital, transported frozen, and extracted at BGI-Shenzhen as Qin 2012 [33] described.

(3). Fresh faecal samples were obtained at home, and samples were immediately frozen by storing in a home freezer for less than 1 day. Frozen samples were transferred to BGI-Shenzhen, and then stored at -80 °C until analysis.

### Analysis of the differences in microbial communities among BMI groups

**Fig. 1:**
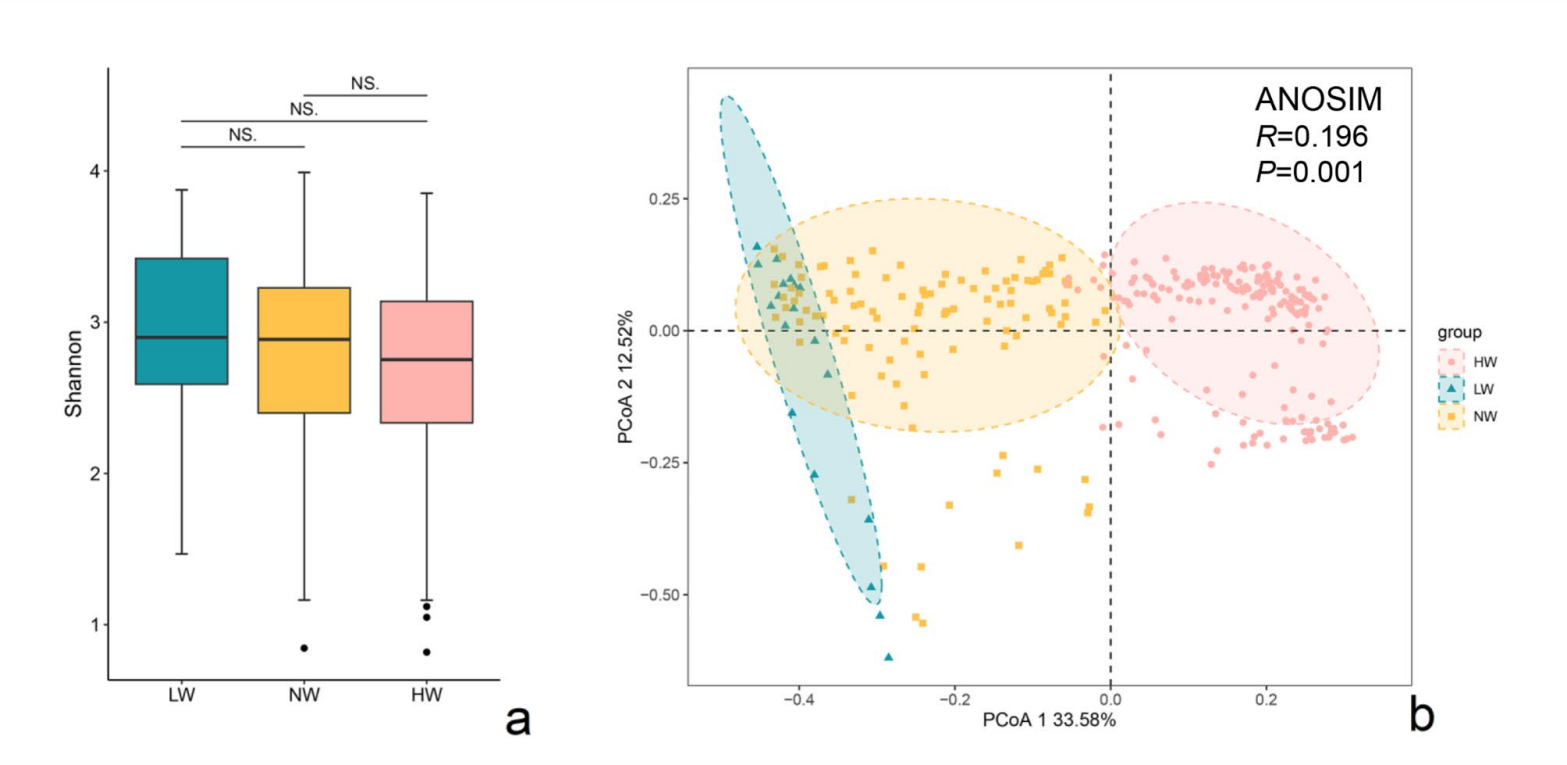
Comparison of gut microbial diversity at the species level. The Shannon index represents α-diversity, and principal coordinate analysis (PCoA) based on the Bray‒Curtis distance represents β-diversity. The overweight group is designated pink, the normal weight group is designated yellow, and the underweight group is designated green (Wilcox test, NS: *p* ≥ 0.05, *: *p* < 0.05, **: *p* < 0.01, ***: *p* < 0.001)

Alpha diversity was used to indicate changes in the overall gut microbial community across the three BMI groups. The Shannon index (Fig. 1a) and Ace index (Additional file 1: Figure S1a) were higher in the underweight group and lower in the overweight group than in the other groups. However, there was no significant difference in α diversity between the BMI groups. Beta diversity was measured with Bray‒Curtis distance and visualized with PCoA (*p* = 0.001). The classification between the normal weight and overweight groups, as well as between the underweight and overweight groups, showed significant differences (Fig. 1b). Most samples clustered within their own groups, implying that the overweight group had a different overall gut microbial community structure than the other two groups. Moreover, most of the samples in the underweight group formed a cluster with the normal weight group, and no clear distinction was observed between them.

**Fig. 2:**
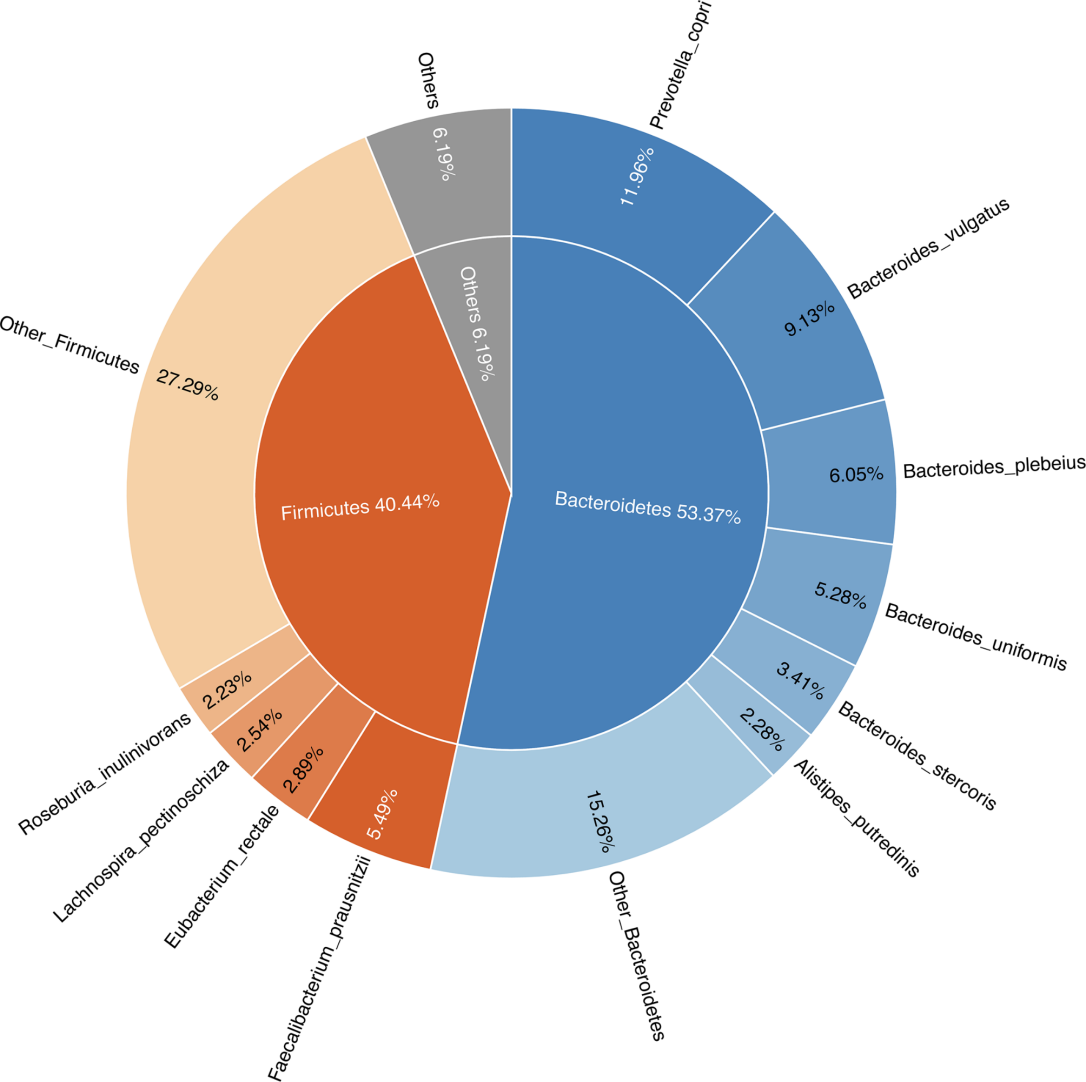
Species composition in all samples. (i) Inner: pie chart of phylum composition of all samples. (ii) Outer: a doughnut chart of species composition in all samples, with the top ten species listed. All species belonging to the same phylum are grouped and labeled with a gradient of color for the corresponding phylum in the inner pie chart. The names and proportions of each species are marked in the corresponding positions. *Prevotella copri* and *Bacteroides vulgatus* were the most common species in the intestine

Differences in the composition of gut microbial communities were analyzed. The fecal samples contained 12 phyla and 735 species. In each BMI group, the major phyla of the bacterial community were *Bacteroidetes*, *Firmicutes*, *Proteobacteria* and *Actinobacteria*. The sum of the abundances of *Bacteroidetes* and *Firmicutes* accounted for more than 90% of the total (Fig. 2). The abundance of *Bacteroidetes* in the normal weight group was lower than that in the other two groups, and the abundance of *Firmicutes* was lower than that in the normal weight group in both the underweight and overweight groups, but no significant difference was observed between the groups. The abundance of *Actinobacteria* showed a significant difference between the underweight group and both the overweight and normal weight groups. The ratio of *Firmicutes* to *Bacteroidetes* (F/B) was calculated and was lower in the underweight and overweight groups than in the normal weight group. Correlation analyses between the F/B ratio and BMI showed no meaningful associations (Additional file 1: Figure S1c). Differences in taxonomic units in the gut microbiota at the species level were assessed. The most abundant species were *Prevotella copri*, *Bacteroides vulgatus*, *Bacteroides uniformis*, *Bacteroides plebeius*, *Faecalibacterium prausnitzi*, *Bacteroides stercoris*, *Bacteroides dorei*, *Eubacterium rectale*, *Lachnospira pectinoschiza*, and *Alistipes putredinis*, as shown in Fig. 2.

**Fig. 3:**
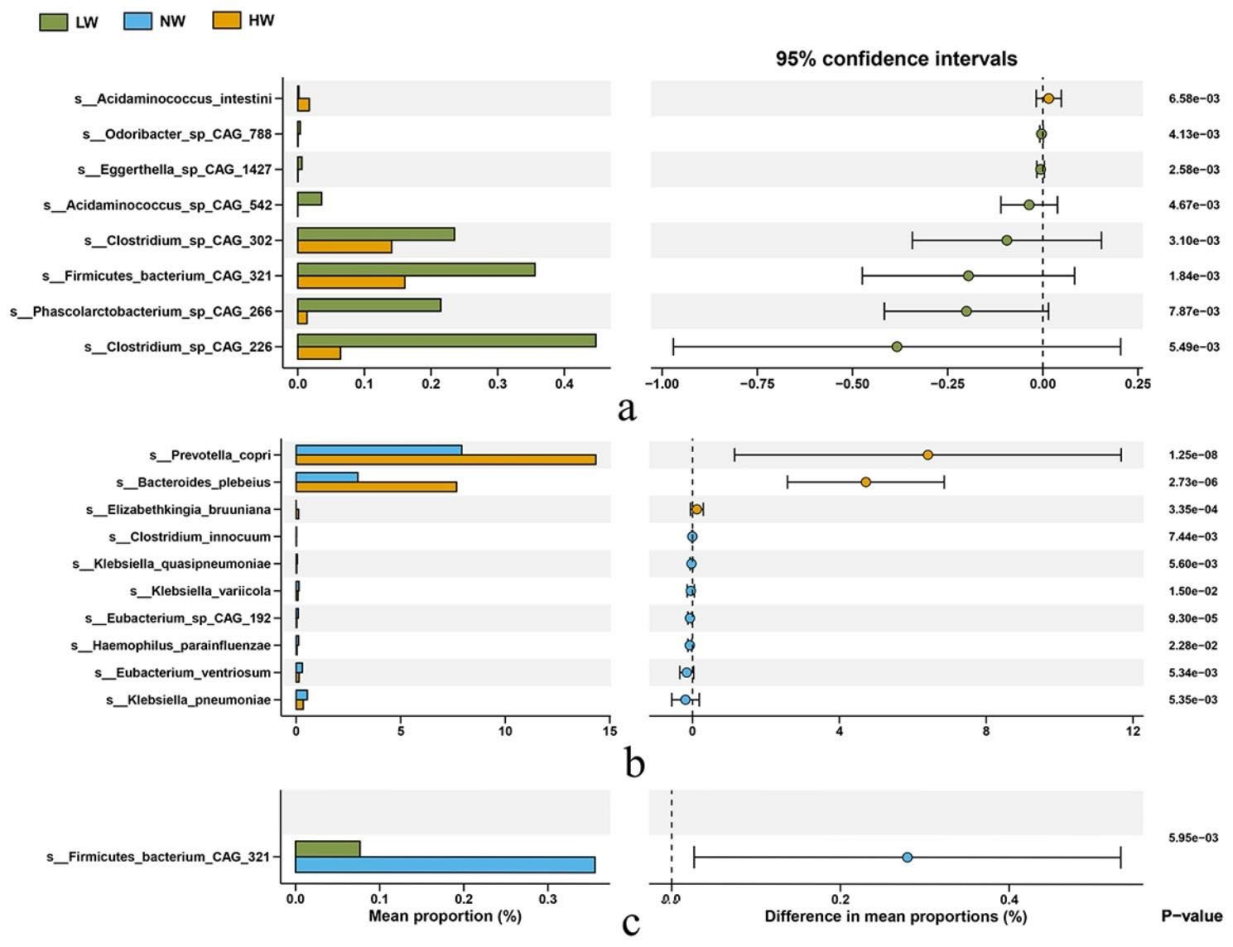
Comparison of gut metagenomes of different BMI groups at the species level. Significant differences in the relative abundance of species were observed between BMI groups (*p* < 0.05). HM: overweight group; NM: normal weight group; LM: underweight group

Figure 3 illustrates differences in gut microbial communities between each BMI group, with a total of 19 species exhibiting significant differences between the groups. Participants in the overweight group had a significant increase in the abundance of one species, and those in the underweight group had a significant increase in the abundance of seven species, as shown in Fig. 3a. The comparison between the overweight group and normal weight group revealed a statistically significant increase in the abundance of three species in the overweight group and seven species in the normal weight group (Fig. 3b). In contrast, the analysis of the difference between the normal weight group and the underweight group revealed a significant increase in the abundance of only one species in the normal weight group (Fig. 3c).

**Fig. 4:**
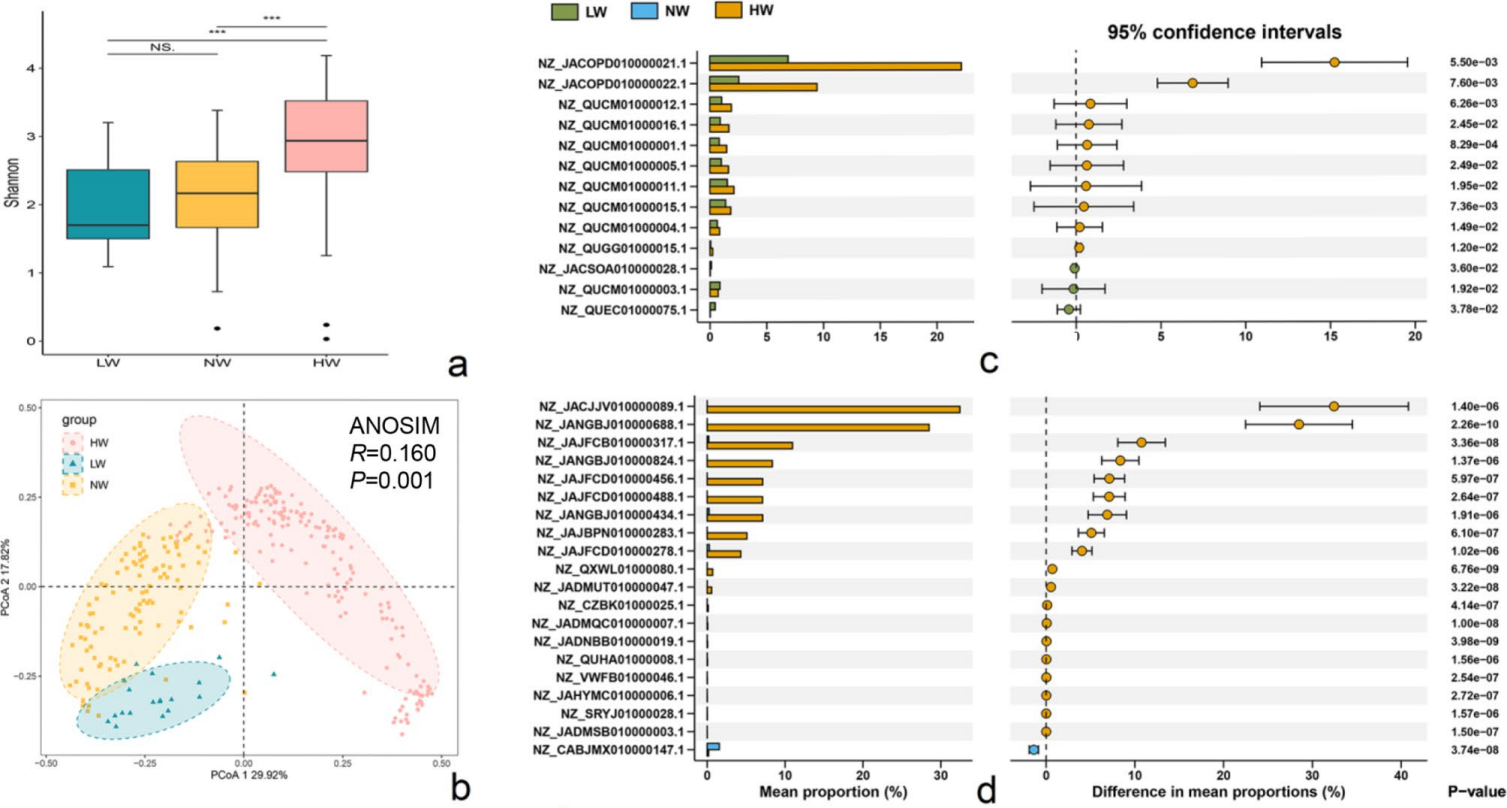
Differential analysis of gut microbiota in various BMI groups at the gene level. (**a**) The Shannon index shows α-diversity. (**b**) Principal coordinate analysis (PCoA) based on the Bray‒Curtis distance shows β-diversity (Wilcox test, NS: *p* ≥ 0.05, *: *p* < 0.05, **: *p* < 0.01, ***: *p* < 0.001). (**c**) (**d**) Contigs with significant differences in SNP density between different BMI groups. HM: overweight group; NM: normal weight group; LM: underweight group

Metagenomic analysis offers the advantage of examining the gut microbiota at the gene level to study its connection to the host. Previous studies have analyzed the genetic diversity in the gut microbiota of healthy and nonhealthy participants in terms of the number of genes with changes at the gene level [34]. We refer to this literature to examine the correlation between gut microbiota and host BMI based on the density of SNPs in the contig with changes at the gene level. First, we analyzed the overall changes in the gene levels of gut microbiota in each BMI group. The Shannon index (Fig. 4a) and Ace index (Additional file 1: Figure S1b) of the overweight group were significantly higher than those of the other two groups. Of note, the difference between the overweight and normal weight groups was the most significant. In summary, the α diversity of the overweight group was the highest among the three groups, which is different from our previous findings regarding species abundance. The PCoA revealed clear differentiation between each BMI group (*p* = 0.001), with the samples clustering separately within the groups. Only a small number of overweight samples clustered in the normal weight group, as shown in Fig. 4b. Differences in gut microbiota gene levels were assessed between BMI groups. Among the total of 372 contigs with significant differences between groups (Additional file 4), we observed a significant increase in SNP density for 10 contigs in overweight participants and 3 contigs in underweight participants when comparing the overweight and underweight groups (Fig. 4c). Although there was a significant difference of 359 contigs in SNP density between the overweight and normal weight groups, we restricted our analysis to the top 20 contigs showing differences. We observed a significant increase in the SNP density of 19 contigs for overweight participants and 1 contig for normal weight participants (Fig. 4d). Our results revealed that there were significantly more genetic trait differences than community trait differences between BMI groups.

### Building prediction models

#### Building three BMI prediction models

**Fig. 5:**
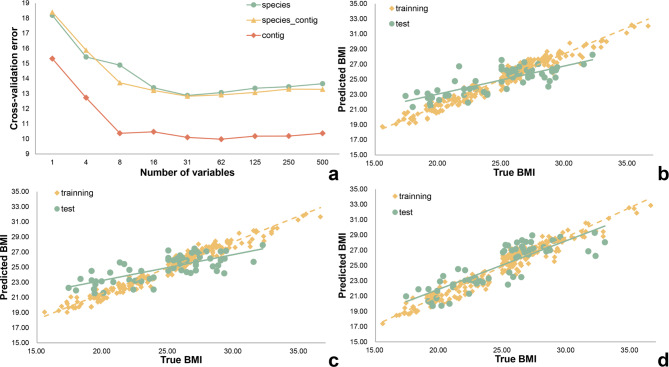
The prediction of BMI by random forest regression analysis. (**a**) Model cross-validation error rate. (**b**) Species abundance regression model results with an R^2^ of 0.56 and an MAE of 2.09. (**c**) Species abundance and single-sample SNP density regression model results with an R^2^ of 0.62 and an MAE of 2.06. (**d**) Contig SNP regression model results with an R^2^ of 0.72 and an MAE of 1.56. The green line represents the species level, the yellow line accounts for both the species and gene level, and the orange‒red line pertains solely to the gene level

All data were randomly divided into an 80% training set and a 20% test set. A model for predicting BMI based on gut microbiota was built by regressing the relative abundance of gut microbiota on BMI at the species level. The test set of the prediction model had an R^2^ value of 0.64 and an MAE of 2.15 kg/m^2^. To minimize prediction errors and exclude biomarkers that do not impact or negatively affect the model, we employed tenfold cross-validation to evaluate the importance of bacterial taxa as biomarkers in predicting BMI. Our cross-validation analysis demonstrated that the model’s prediction error significantly decreased with an increase in the number of species used until it exceeded 31 (Fig. 5a). The 31 most important species are displayed in Additional file 1: Figure S2. In the BMI prediction model, *Bacteroides plebeius* was the most important species, followed by *Prevotella copri*, *Elizabethkingia bruuniana*, and *Clostridium* sp. *AM22_11AC*. Using these 31 biomarkers, we developed a new BMI prediction model with an R^2^ of 0.56 and an MAE of 2.09 kg/m^2^, as shown in Fig. 5b.

This paper demonstrates that genetic characterization of the gut microbiota is more stable than taxon abundance and may be a more effective predictor of individual traits. We used SNP densities to represent changes at the genetic level of the gut microbiota. Total SNP density was calculated for each sample using Python, and it was combined with species abundance as a metric for predictive modeling. Tenfold cross-validation was used to minimize cross-validation error while selecting the 31 biomarkers that contributed the most to the regression model (Fig. 5a). The R^2^ of the model test set was 0.62 with an MAE of 2.06 kg/m^2^ (Fig. 5c). The species that most contributed to the predictive model was *Prevotella copri*, followed by *Bacteroides plebeius* and *Elizabethkingia bruuniana*. The fourth highest contribution was the individual sample SNP density. The inclusion of genetic indicators resulted in a slight improvement in the model’s accuracy, suggesting that genetic characteristics of the gut microbiota may be more effective in predicting actual BMI values.

Therefore, the SNP density of each contig in all samples was obtained through Python computation and used as a biomarker to construct an individual BMI prediction model with the RF machine learning algorithm. The R^2^ of the model test set was 0.73 with an MAE of 1.52 kg/m^2^. Through tenfold cross-validation, we determined that the prediction model constructed with 62 contigs had the lowest error (Fig. 5a). Information on the species corresponding to the top 62 most significant contigs was obtained. The analysis revealed that the top five significant contigs were two from *Parabacteroides distasonis* and three from *Lachnospira eligens*. *Prevotella copri* exhibited the highest contig count, with 17. The selected contigs were used to reconstruct the predictive BMI model. The ’R^2^ of the model was 0.72 with an MAE of 1.56 kg/m^2^ (Fig. 5d).

#### Prediction of three body types (LW, NW, HW)

**Fig. 6:**
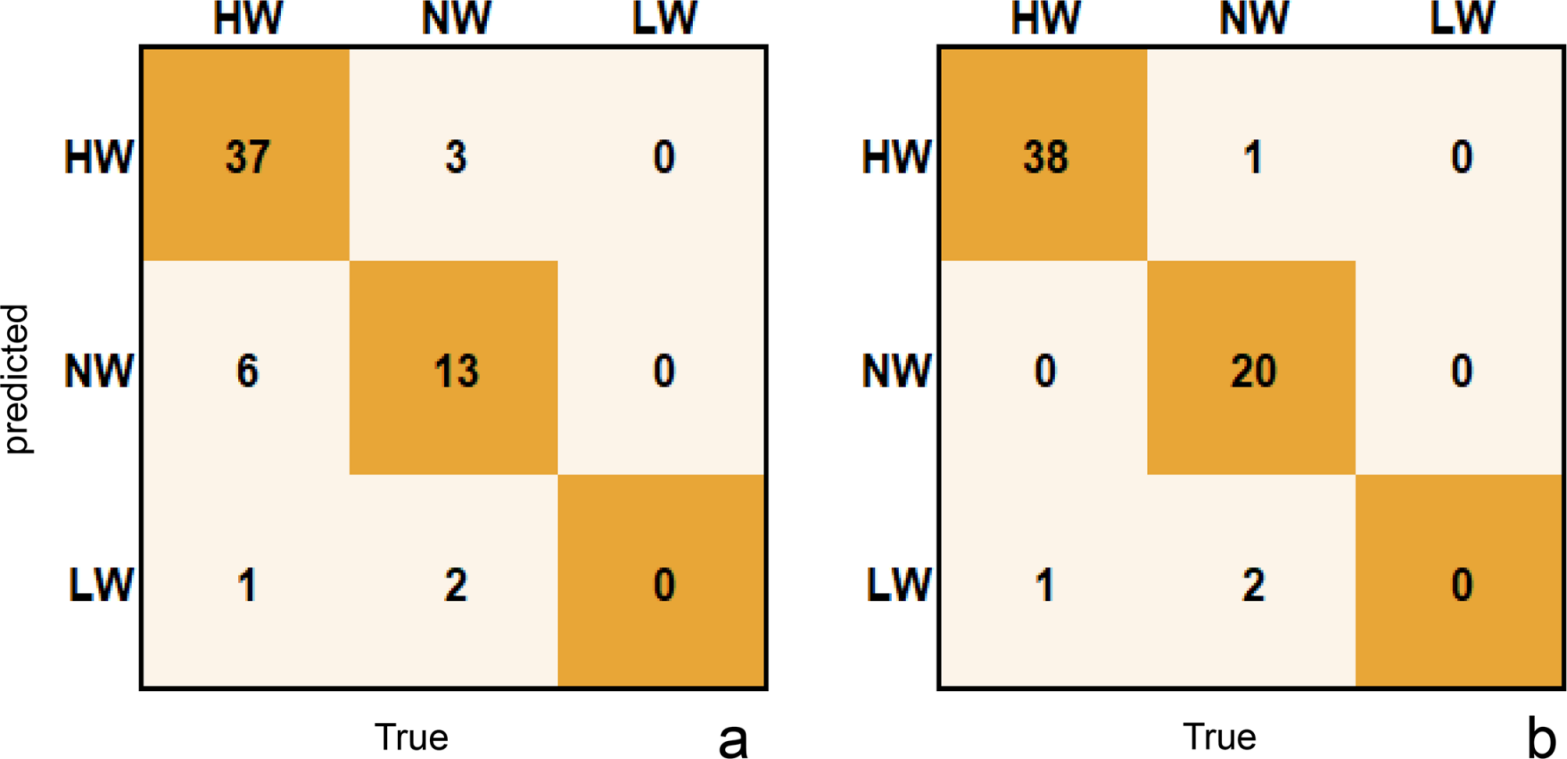
The prediction of body type by random forest classification analysis. **a** is the species abundance model, and 80.65% of the individuals were correctly predicted. **b** is the contig SNP density model, and 93.55% of the individuals were correctly predicted. HM: overweight group; NM: normal weight group; LM: underweight group

Using the RF classification algorithm and based on the two biomarkers of species relative abundance and contig SNP density, we built separate body type prediction models. All data were randomly divided into an 80% training set and a 20% test set. Through tenfold cross-validation, 44 imperative biomarkers were selected by the two classification models. Interestingly, 70% of these biomarkers were also among the top markers identified by the BMI prediction model. A total of two confusion matrices (Fig. 6) were obtained. The accuracy of predicting body shape based on the species abundance index in the test set was 80.65% (Fig. 6a), while the accuracy of predicting body shape based on the SNP density index was as high as 93.55% (Fig. 6b). The accuracy of the latter body shape prediction was significantly higher than that of the former. Moreover, the prediction error rate decreased for both the overweight and normal weight groups, with 100% of the samples in the normal weight group being predicted correctly. Our results are consistent with previous findings for BMI prediction, which indicated that the SNP density model was the most accurate predictor.

## Discussion

The gut microbial community is closely related to human health, as it is the largest and most complex microbial system in the human body. The number of microbial cells in the human gut is ten times greater than the number of cells in the human body [35]. The large number of gut microbiota contains much more information than traditional human DNA genetic markers. However, effective methods for interpreting and analyzing this information are currently lacking. For instance, in some specific real-life cases, feces may be the only valuable physical evidence that remains at the crime scene. However, identifying human DNA in feces is challenging due to inhibitors, degradation, and low concentrations of template DNA [36]. The gut microbiota may aid in locating suspects if it can be used to accurately infer their personal characteristics. Thus, the gut microbiota has potential as a promising new direction for solving challenging cases or as a crucial clue in unique circumstances.

Most current research on individual characteristics such as age [37] and body type [16] based on microbiome information has focused on the species level. A commonly used strategy is to collect species data from all samples using second-generation sequencing technology. Here, the α-diversity and β-diversity of microbial communities were then analyzed based on species abundance to explore intra- and interindividual diversity. Differences in microbial community composition and abundance between groups were also analyzed, and a model to predict individual characteristics was created using taxa with significant differences. However, it has been shown that microbial gene-level variation is also closely linked to individual traits. Genetic traits at the gene level are more stable over time and more specific to each individual than the diversity of the gut microbial community [18].

Therefore, the aim of this study was to establish the correlation between species community characteristics, genetic traits and BMI. We assessed the α and β diversity of gut microbial communities at the species and gene levels to reflect changes in microbial communities. Whether there were differences in microbial diversity within and between groups at the species and gene levels was explored. We analyzed the major bacteria at the species level and compared them with previous studies to identify similarities and differences. Specific bacterial and gene ANOVAs were performed at the species and gene level to examine the bacteria and genes that showed significant differences within each BMI group. These findings provide a theoretical basis for developing predictive models. Finally, we constructed an RF model using selected biomarkers and compared the accuracy of the species and gene models in predicting BMI and body type.

Using the same sequencing platform can minimize systematic errors in the analysis. We identified healthy Chinese populations from the database to serve as study participants based on gut metagenomic data obtained through the Illumina sequencing platform. Furthermore, the phenotypic information of each sample corresponding to the database must be clearly stated in the literature. Subject to the strict screening conditions described above, a total of 308 fecal samples from healthy Chinese volunteers with metagenomic data were included in the study. Body type can serve as a descriptive data point for suspects’ appearances, allowing forensic scientists to limit the pool of suspects. Recent studies have demonstrated a correlation between body type and the composition of the gut microbial community [38–41]. We examined the gut microbial community diversity in various BMI groups and discovered that α-diversity declines with increasing BMI, while not having significant differences between each BMI category. This suggests that the richness and evenness of gut microbes tend to decline with increasing BMI. A study [16] that examined gut microbiota diversity to predict BMI in Chinese university students displayed findings similar to ours. Specifically, BMI demonstrated a slightly negative association with gut microbiota α-diversity, as supported by a Pearson correlation. Other studies with similar subgroup types to ours have shown mutually inconsistent results [42, 43]. Yun et al. [43] found that the overall α-diversity of study participants significantly decreased as BMI increased in a large Korean cohort based on a comparative analysis of the gut microbiota in relation to BMI. According to a Japanese study [42], microbial diversity and richness were significantly higher in obese volunteers than in nonobese volunteers. This could be attributed to the influence of external factors on gut microbes, such as place of residence [44], health status [45], and mental stress [46]. Furthermore, a study indicated that there are differences in the gut microbiota among various populations in the United States, possibly due to cultural dietary adaptations [47]. Despite being Asian countries, China, Japan, and Korea have different dietary cultures that could explain the inconsistent results in the aforementioned studies.

Obesity is associated with an abundance of *Firmicutes* and *Bacteroidetes*, as well as the F/B ratio, as demonstrated by numerous studies. In our study, overweight participants had a lower abundance of *Firmicutes*, higher abundance of *Bacteroidetes*, and a reduced F/B ratio compared to normal weight participants. However, these changes were not statistically significant, which is in line with the results of a large cohort study in Korea [43]. Ley RE et al. reported that the obese population had higher levels of *Firmicutes* and lower levels of *Bacteroidetes* than the normal population, whereas Schwiertz A et al. demonstrated a significant decrease in *Firmicutes* and a significant increase in *Bacteroidetes* in the obese population, resulting in a significant decrease in the F/B ratio [48, 49]. The discrepancies in the aforementioned findings could be attributed to the complex and diverse factors affecting the composition of the gut microbial community, including sex and age [50]. Our study was specifically designed to address the complexity and variability of forensic scene conditions. As such, we did not artificially control for age and sex variables when analyzing the data. This is in contrast to other studies where the distribution of age ranges and sex ratios among volunteers varied. Furthermore, the accuracy of the results in a study is highly dependent on the sample size. An inadequate sample size may result in insignificant differences within and between groups, thereby affecting the categorization of individuals based on their body type. For example, the previous studies [42, 48, 49] had a sample size of only a few dozen or even a dozen subjects, whereas our study with Yun et al. [43] analyzed more than 300 subjects. Several other studies have demonstrated the influence of factors such as the geographic origin [51]and dietary habits [52] of the population on the composition and diversity of the gut microbial community in both the short and long term. The participants in this study were from different provinces in China, each with its own set of dietary habits and preferences. This might explain the variations in BMI-related gut microbiota composition and diversity among different BMI groups in various studies.

Compared to 16 S rRNA, second-generation metagenomic sequencing offers deeper sequencing depth, higher annotation accuracy at the species level, and a more comprehensive analysis at the gene level. In this study, *Prevotella copri* was the most abundant species in the three BMI groups. The overweight group had a significantly higher relative abundance of *P. copri* than the normal weight group. Conversely, *Bacteroides uniformis* had a significantly lower relative abundance in the overweight group than in the normal weight group. Duan et al. conducted a study in a Han Chinese population in northern China that showed a phenomenon consistent with ours [53]. Vallianou et al. reported that *B. uniformis*, as a next-generation probiotic (NGP), had beneficial properties against obesity and was associated with weight loss in animal models [54]. This finding is consistent with the significant reduction in *B. uniformis* observed with increasing BMI in the present study. Our analysis showed that the abundance differences between the BMI groups at the species level significantly distinguished the overweight group from the normal weight group, which may explain why the two sample groups are easily distinguished in the PCoA analysis.

Forensic scientists currently utilize microorganisms to deduce individual characteristics by primarily examining the structure and abundance of microbial communities, i.e., by focusing on the presence and quantity of microorganisms. Nonetheless, studies have demonstrated that changes at the microbial genome level are closely linked to individual characteristics. In their research, Li et al. [55] observed that a high-fiber diet heightened the SNP proportions of *Faecalibacterium*, *Bifidobacterium*, and *Clostridium* while reducing those of *Bacteroides* in obese children. According to Chen et al. [18], the accuracy of categorizing two samples as belonging to the same individual based on species abundance was only 12%. In contrast, the classification accuracy significantly increased to 94% when using genetic characteristics such as SNP haplotypes and SVs. This indicated that the genetic characteristics of gut microorganisms are more stable and individual specific, making them useful as ‘microbial fingerprints’ for profiling personal characteristics. Consequently, the changes at the microbial genome level should not be dismissed. To account for the effect of fragment length on the number of SNPs, we compared fragments of equal length and expressed the results in terms of the density of gut microbial SNPs, which represents the number of SNPs per kilobase of DNA. Our study found inconsistencies between the gene levels of gut microbial communities and the taxonomic levels. Specifically, we observed a significant difference in the α-diversity of SNP density between BMI groups. The α-diversity of the overweight group was appreciably higher than that of the other groups (p < 0.001). In addition, both the richness and evenness of gut microbial genes revealed a significant positive trend with increasing BMI. Samples from each BMI group were clustered significantly in PCoA. This phenomenon suggests that there are significant characteristic differences among the bacteria and genes of different BMI groups, which effectively differentiates the groups and provides the necessary theoretical support for the body type prediction model that follows. In addition, our results showed that there were 19 biomarkers with significant differences at the species community level between each BMI group and 372 biomarkers with significant differences at the gene level. This demonstrated that more characteristics were measurably linked to BMI at the gene level, indicating a stronger correlation between genetic traits and BMI compared to community traits. These significant differences could be helpful in inferring BMI from the perspective of species genes, which will provide a more accurate method for inferring individual characteristics.

Advances in high-throughput sequencing technology have increased the amount of microbial information available to forensic scientists. This information can be used to differentiate between individuals or groups based on the abundance of species present in the gut microbial community or to infer various traits to aid in investigation and trial evidence. Wang et al. [16] developed a regression model based on 44 unique genera in three BMI groups to investigate the association between BMI and the gut microbiome. This model proved successful in correctly predicting body shape in 74% of the samples. This indicates the possibility of distinguishing between individuals using bacteria with significant variation. To determine whether genetic or community characteristics of bacteria are more effective predictors of individual traits, we constructed two RF regression models. The first was based on the relative abundance of species, and the second was based on the SNP density of contigs. Both models were used to predict BMI. The regression model, which was based on the importance ranking of the top 31 species, had an R^2^ value of 0.56 and an MAE of 2.09 kg/m^2^, which is similar to the regression analysis results of Wang et al.^16]^. Following this, we included the SNP density of individual samples as a biomarker to the model, which led to better predictive performance compared to the model based only on species abundance. Specifically, the R^2^ value increased while the MAE decreased, and the SNP density index of individual samples played a significant role in improving model performance, suggesting that genetic characteristics of gut bacteria may be more effective in predicting individual BMI. To investigate this, we measured the SNP densities of all sample contigs and used the Spearman test to determine the correlation between species abundance and contig SNP density with BMI. Our findings indicate that there is a low and almost statistically nonsignificant correlation between species abundance and BMI. On the other hand, the correlation between SNP densities of the 767 contigs is relatively high, which may suggest that genetic traits are more influential than community traits. Based on these findings, the SNP densities of 62 contigs were chosen as indicators to create the prediction model, and we achieved the best fit and accuracy with an R^2^ value of 0.72 and an MAE of 1.56 kg/m^2^. These results highlighted the effectiveness of the BMI prediction model based on contig SNP density, which demonstrated the highest accuracy and the lowest mean absolute error. In conclusion, the findings indicate that the genetic specificity of gut bacteria is a more reliable predictor of individual characteristics than community specificity, and using genetic characteristics of gut bacteria to predict individual BMI is an innovative and effective approach that requires further research.

To further validate the utilization of gut bacterial genetic specificity for the prediction of individual traits over community specificity, we established a model for the prediction of body shape based on two markers, species abundance and contig SNP density. The SNP density model showed 94% accuracy in predicting body shape, which was significantly higher than that of the species abundance model. However, all the underweight samples were inaccurately predicted by both models. This might be due to the limited number of underweight volunteers who participated in our study, and their BMI was close to normal weight, which averaged 17.60 (± 0.47), making it impossible to correctly characterize the samples in the underweight group. The comparable gut microbial composition in the underweight and normal weight groups may also be a plausible explanation. Our study showed a significant difference in the abundance of just one species between the two groups, but none of the contigs’ SNP densities varied significantly. Furthermore, Wang et al. [16] used a linear regression model to estimate body type; however, only one sample from the underweight group was accurately predicted. This observation also highlights the challenge of distinguishing between the underweight and normal weight groups and needs to be addressed in future studies.

This study presents an initial investigation into gut metagenomes and genetic characterization of microorganisms as they relate to forensic practice. Examination of gut metagenomes allows for the investigation of potential correlations between bacteria and hosts, such as specific species and gene-level associations. The genetic characterization of gut microbes can predict the individual’s BMI and body type with higher accuracy than the community characterization of species. This implies that the genetic attributes of bacteria can be utilized to infer individual characteristics with precision. In this study, the correlation between the gut microbiome and human BMI were examined in a healthy Chinese population. It must be noted that an individual’s smoking status, alcohol consumption, and medication history are typically unknown in forensic cases, as is their health status and whether they suffer from any diseases. Future studies should take into account a broader range of factors that affect the gut microbiome. In conclusion, the applicability of the gut microbiome in forensic personal characterization requires further research.

## Conclusion

In this study, the association between changes in the microbial community and gene levels and host BMI characteristics was investigated using gut metagenomic data. We utilized the RF machine learning algorithm to develop two prediction models, and these models exhibited high accuracy on both the training and test sets, indicating a significant correlation between the gut microbiome and personal BMI characteristics. In particular, the prediction model based on species SNP density delivered the best performance: the model accurately predicted BMI with an R^2^ of 0.72 and an MAE of 1.56 kg/m^2^, and the accuracy of predicting body type was 94%. This corroborates the potential of forensic BMI or body type trait inference at the genome level, offering novel ideas and methods for practical forensic applications to deduce external features of criminal suspects.

### Electronic supplementary material

Below is the link to the electronic supplementary material.

Supplementary Material 1

## Data Availability

The paper’s findings are based on data from the NCBI Sequence Reads Archive, obtained through accession numbers PRJNA539850, SRA045646, and PRJEB6997. (https://www.ncbi.nlm.nih.gov/bioproject/PRJNA539850). (SRA045646 - SRA - NCBI (nih.gov)). (https://www.ncbi.nlm.nih.gov/bioproject/PRJEB6997).

## References

[1] Wang SS, Song F, Wei X (2022). Research Progress on the application of human oral microbiome in forensic individual Identification[J]. Fa Yi Xue Za Zhi.

[2] Giurazza F, Del Vescovo R, Schena E et al. Determination of Stature from Skeletal and Skull Measurements by Ct Scan Evaluation[J]. Forensic Sci Int, 2012, 222(1–3): 398 e391-399. 10.1016/j.forsciint.2012.06.008 22749675

[3] Macaluso PJ, Lucena J (2014). Stature estimation from Radiographic Sternum length in a Contemporary Spanish Population[J]. Int J Legal Med.

[4] Pininski M, Brits D (2014). Estimating stature in South African populations using various measures of the Sacrum[J]. Forensic Sci Int.

[5] Chevalier T, Lefevre P, Clarys JP (2016). The Accuracy of Body Mass Prediction for Elderly specimens: implications for Paleoanthropology and Legal Medicine[J]. J Forensic Leg Med.

[6] Lacoste Jeanson A, Santos F, Villa C (2017). Body Mass Estimation from the Skeleton: an evaluation of 11 Methods[J]. Forensic Sci Int.

[7] Lorkiewicz-Muszynska D, Przystanska A, Kociemba W (2013). Body Mass Estimation in Modern Population using anthropometric measurements from computed tomography. Forensic Sci Int.

[8] Liu F, Zhong K, Jing X (2019). Update on the predictability of Tall stature from DNA markers in Europeans[J]. Forensic Sci Int Genet.

[9] Jiao HY, Sun YN, Jing XX (2018). [Assessment of Height Prediction Model based on snps loci][J]. Fa Yi Xue Za Zhi.

[10] Armet AM, Deehan EC, O’Sullivan AF (2022). Rethinking healthy eating in light of the gut Microbiome[J]. Cell Host Microbe.

[11] Tilg H, Kaser A (2011). Gut microbiome, obesity, and metabolic Dysfunction[J]. J Clin Invest.

[12] Vasanth Rao VRB, Candasamy M, Bhattamisra SK, Diabetes. & Metabolic Syndrome: Clinical Research & Reviews, 2019, 13(3): 2112–20. 10.1016/j.dsx.2019.05.004 31235145

[13] Wang Y, Beydoun MA (2007). The obesity epidemic in the United States gender, age, socioeconomic, Racial/Ethnic, and Geographic characteristics: a systematic review and Meta-regression Analysis[J]. Epidemiol Rev.

[14] Sinha R, Jastreboff AM (2013). Stress as a common risk factor for obesity and Addiction[J]. Biol Psychiatry.

[15] Gao X, Zhang M, Xue J (2018). Body Mass Index differences in the gut microbiota are gender Specific[J]. Front Microbiol.

[16] Wang S, Song F, Gu H (2022). Assess the diversity of gut microbiota among healthy adults for forensic Application[J]. Microb Cell Fact.

[17] Temperton B, Giovannoni SJ (2012). Metagenomics: Microbial Diversity through a scratched Lens[J]. Curr Opin Microbiol.

[18] Chen L, Wang D, Garmaeva S (2021). The Long-Term Genetic Stability and Individual specificity of the human gut Microbiome[J]. Cell.

[19] Schloissnig S, Arumugam M, Sunagawa S (2013). Genomic Variation Landscape of the human gut Microbiome[J]. Nature.

[20] Pubmed. https://Pubmed.Ncbi.Nlm.Nih.Gov/ [J]. Accessed 1 Jul 2021.

[21] Abouelkhair MA (2020). Non-sars-cov-2 genome sequences identified in clinical samples from Covid-19 infected patients: evidence for Co-Infections[J]. PeerJ.

[22] Chen Y, Li Z, Hu S (2017). Gut metagenomes of type 2 Diabetic patients have characteristic single-nucleotide polymorphism distribution in Bacteroides Coprocola[J]. Microbiome.

[23] Fastqc SA. A Quality Control Tool for High Throughput Sequence Data[J]. 2010.

[24] Chen S, Zhou Y, Chen Y (2018). Fastp: an Ultra-fast All-in-one Fastq Preprocessor[J]. Bioinformatics.

[25] Beghini F, McIver LJ, Blanco-Miguez A et al. Integrating Taxonomic, Functional, and strain-level profiling of Diverse Microbial communities with Biobakery 3[J]. Elife, 2021, 10. 10.7554/eLife.65088 PMC8096432 33944776

[26] Li H. Aligning sequence reads, clone sequences and Assembly contigs with Bwa-Mem[J]. arXiv preprint; 2018.

[27] Li H, Handsaker B, Wysoker A (2009). The sequence Alignment/Map format and Samtools[J]. Bioinformatics.

[28] Picard. http://Broadinstitute.Github.Io/Picard/ [J]. Accessed 20 Sept 2021.

[29] Danecek P, Auton A, Abecasis G (2011). The Variant Call Format and Vcftools[J] Bioinformatics.

[30] Liaw A, Wiener M. Classification and regression by Randomforest[J]. R News, 2002, 2 (3).

[31] Zeng Q, Yang Z, Wang F et al. Association between Metabolic Status and Gut Microbiome in obese Populations[J]. Microb Genomics, 2021, 7(8). 10.1099/mgen.0.000639 PMC8549370 34356001

[32] Zhang X, Zhang D, Jia H (2015). The oral and gut microbiomes are perturbed in rheumatoid arthritis and partly normalized after Treatment[J]. Nat Med.

[33] Qin J, Li Y, Cai Z (2012). A Metagenome-Wide Association Study of Gut Microbiota in type 2 Diabetes[J]. Nature.

[34] Zhu F, Ju Y, Wang W (2020). Metagenome-Wide Association of Gut Microbiome Features for Schizophrenia[J]. Nat Commun.

[35] Suau A, Bonnet R, Sutren M et al. Direct analysis of genes encoding 16s Rrna from Complex communities reveals many novel molecular species within the human Gut[J]. Appl Environ Microbiol 1999 Nov, 65(11):4799–807. 10.1128/aem.65.11.4799-4807.1999 PMC91647 10543789

[36] Fernando P, Vidya TN, Rajapakse C (2003). Reliable Noninvasive genotyping: Fantasy or reality?[J]. J Hered.

[37] Huang S, Haiminen N, Carrieri A-P et al. Human skin, oral, and Gut microbiomes Predict Chronological Age[J]. mSystems, 2020, 5(1). 10.1128/mSystems.00630-19 PMC7018528 32047061

[38] Gomes AC, Hoffmann C, Mota JF (2018). The human gut microbiota: metabolism and perspective in Obesity[J]. Gut Microbes.

[39] Liu R, Hong J, Xu X (2017). Gut microbiome and serum metabolome alterations in obesity and after weight-loss Intervention[J]. Nat Med.

[40] Palmas V, Pisanu S, Madau V (2021). Gut microbiota markers Associated with obesity and overweight in Italian Adults[J]. Sci Rep.

[41] Patterson E, Ryan PM, Cryan JF (2016). Gut microbiota, obesity and Diabetes[J]. Postgrad Med J.

[42] Kasai C, Sugimoto K, Moritani I (2015). Comparison of the gut microbiota composition between obese and non-obese individuals in a Japanese Population, as analyzed by terminal restriction fragment length polymorphism and next-generation Sequencing[J]. BMC Gastroenterol.

[43] Yun Y, Kim HN, Kim SE (2017). Comparative analysis of Gut Microbiota Associated with Body Mass Index in a large Korean Cohort[J]. BMC Microbiol.

[44] He Y, Wu W, Zheng HM (2018). Regional Variation limits applications of healthy gut Microbiome Reference Ranges and Disease Models[J]. Nat Med.

[45] de Vos WM, Tilg H, Van Hul M (2022). Gut Microbiome and Health: Mechanistic Insights[J] Gut.

[46] Femke L, Louis MAA, Johan DS (2008). The role of Microbiota and Probiotics in stress-Induced gastrointestinal Damage[J]. Curr Mol Med.

[47] Peters BA, Yi SS, Beasley JM (2020). Us Nativity and Dietary Acculturation Impact the gut microbiome in a diverse us Population[J]. ISME J.

[48] Ley RE, Turnbaugh PJ, Klein S (2006). Hum Gut Microbes Assoc Obesity[J] Nat.

[49] Schwiertz A, Taras D, Schafer K (2010). Microbiota and Scfa in lean and overweight healthy Subjects[J]. Obes (Silver Spring).

[50] de la Cuesta-Zuluaga J, Kelley ST, Chen Y et al. Age- and sex-dependent patterns of gut microbial diversity in human Adults[J]. mSystems, 2019, 4(4). 10.1128/mSystems.00261-19 PMC6517691 31098397

[51] Mueller S, Saunier K, Hanisch C (2006). Differences in fecal microbiota in different European study populations in relation to age, gender, and Country: a cross-sectional Study[J]. Appl Environ Microbiol.

[52] Beam A, Clinger E, Hao L. Effect of Diet and Dietary Components on the composition of the gut Microbiota[J]. Nutrients, 2021, 13(8). 10.3390/nu13082795 PMC8398149 34444955

[53] Duan M, Wang Y, Zhang Q (2021). Characteristics of gut microbiota in people with Obesity[J]. PLoS ONE.

[54] Vallianou NG, Kounatidis D, Tsilingiris D et al. The role of next-generation probiotics in obesity and obesity-Associated disorders: current knowledge and future Perspectives[J]. Int J Mol Sci, 2023, 24(7). 10.3390/ijms24076755 PMC10095285 37047729

[55] Li H, Zhao L, Zhang M (2021). Gut Microbial Snps Induced by High-Fiber Diet Dominate Nutrition Metabolism and Environmental Adaption of Faecalibacterium Prausnitzii in obese Children[J]. Front Microbiol.

