# Supplementary material for: Prediction of BMI traits in the Chinese population based on the gut metagenome
Source: Microb Cell Fact. 2023 Dec 8;22:250. doi: 10.1186/s12934-023-02255-3 (PMC10704812; doi:10.1186/s12934-023-02255-3)
Supplement: Supplementary file 1 — Supplementary Material 1 [file 12934_2023_2255_MOESM1_ESM.docx]

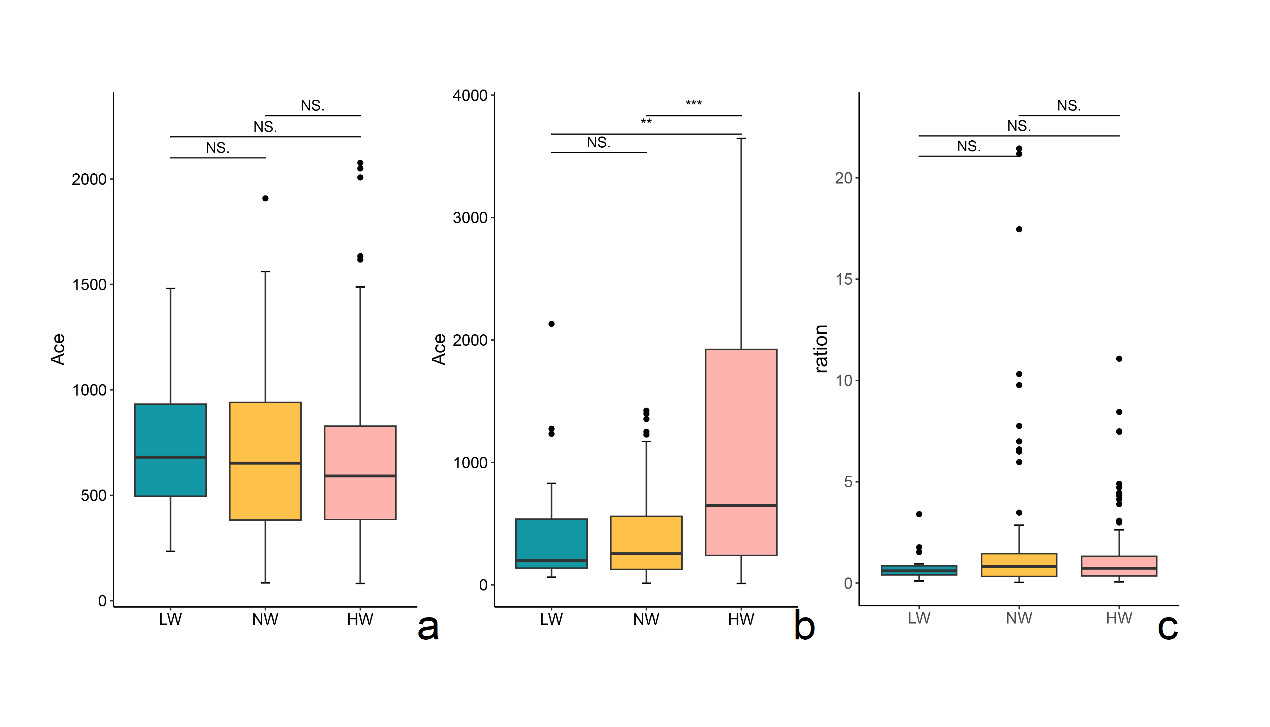


Figure S1：Analysis of α-diversity and Firmicutes/Bacteroidetes ratio. a represents species-level ACE index diversity, while b represents gene-level ACE index diversity. c is Firmicutes/Bacteroidetes ratio. Overweight group is designated by pink, normal-weight group recombination by yellow, and the underweight group by green (wilcox test, NS: p≥0.05, *: p< 0.05, **: p<0.01, ***: p< 0.001).


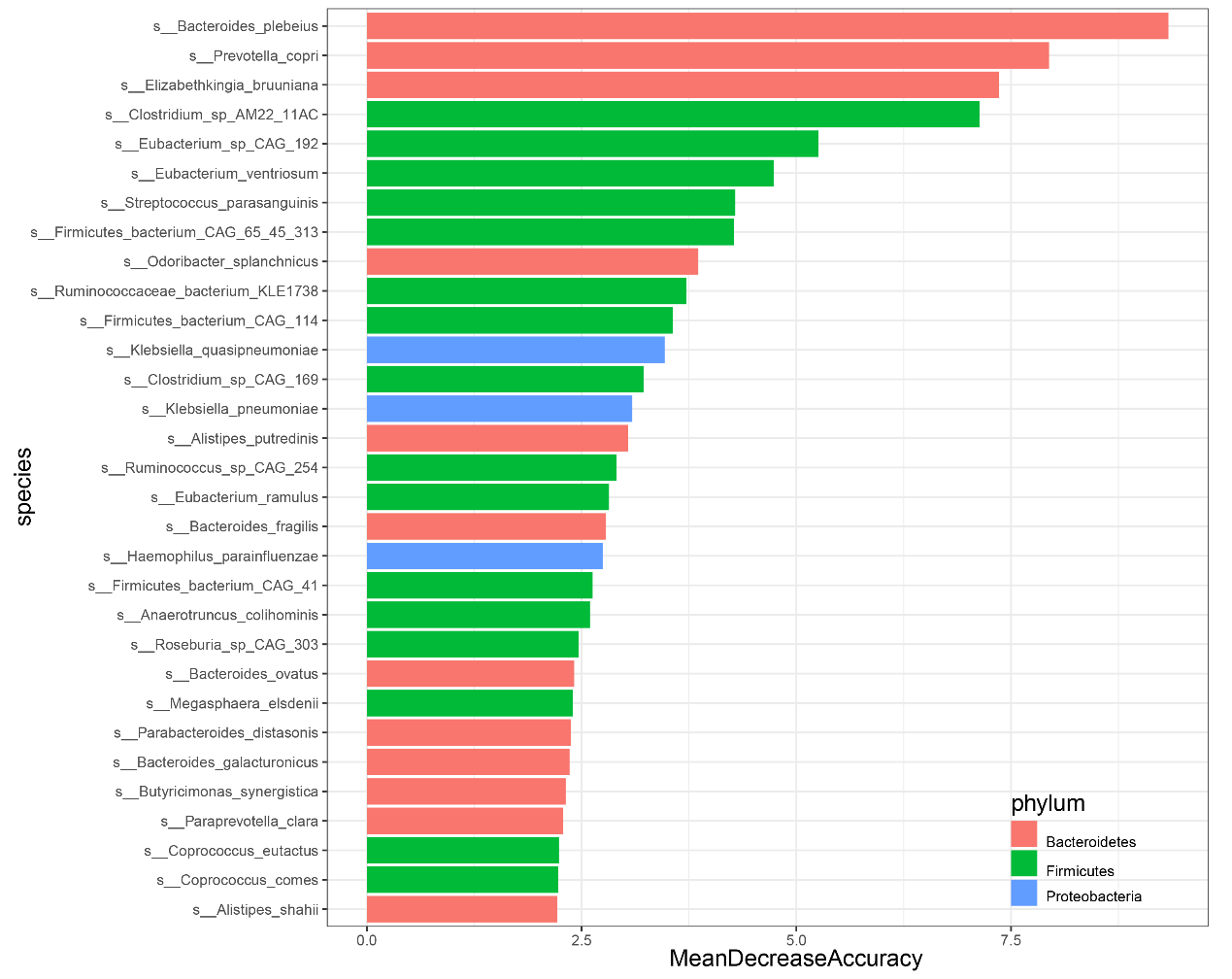
 Figure S2：Ranking of 31 important species. The 31 species screened for the inferential model are displayed on the vertical axis, and the mean decrease accuracy on the horizontal axis. Larger values indicate a greater species importance in the model. *Bacteroidetes* is represented by red color, *Firmicutes* by green, and *Proteobacteria* by blue.
